# Model-Based Quality, Exergy, and Economic Analysis of Fluidized Bed Membrane Reactors

**DOI:** 10.3390/membranes11100765

**Published:** 2021-10-03

**Authors:** Tabassam Nafees, Adnan Ahmed Bhatti, Usman Khan Jadoon, Farooq Ahmad, Iftikhar Ahmad, Manabu Kano, Brenno Castrillon Menezes, Muhammad Ahsan, Naveed ul Hasan Syed

**Affiliations:** 1 Department of Chemical Engineering, National University of Sciences and Technology, Islamabad 44000, Pakistan; mahinmuhammad503@gmail.com (T.N.); rizwanbhatti56@gmail.com (A.A.B.); engrusmanjadoon@gmail.com (U.K.J.); ahsan@scme.nust.edu.pk (M.A.); 2 Department of Chemical and Material Engineering, College of Engineering, Northern Border University, KSA, Arar 9280, Saudi Arabia; famin@nbu.edu.sa; 3 Department of Systems Science, Graduate School of Informatics, Kyoto University, Kyoto 606-8501, Japan; manabu@human.sys.i.kyoto-u.ac.jp; 4 Division of Engineering Management and Decision Sciences, College of Science and Engineering, Hamad Bin Khalifa University, Qatar Foundation, Doha 34110, Qatar; bmenezes@hbku.edu.qa; 5 Department of Chemical Engineering, University of Engineering and Technology, Peshawar 25120, Pakistan; syednaveed@uetpeshawar.edu.pk

**Keywords:** naphtha catalytic reforming, two-phase theory of fluidization, fluidized bed membrane reactor, fluidized bed reactor, exergy analysis, economic analysis

## Abstract

In petroleum refineries, naphtha reforming units produce reformate streams and as a by-product, hydrogen (H_2_). Naphtha reforming units traditionally deployed are designed as packed bed reactors (PBR). However, they are restrained by a high-pressure drop, diffusion limitations in the catalyst, and radial and axial gradients of temperature and concentration. A new design using the fluidized bed reactor (FBR) surpasses the issues of the PBR, whereby the incorporation of the membrane can improve the yield of products by selectively removing hydrogen from the reaction side. In this work, a sequential modular simulation (SMS) approach is adopted to simulate the hydrodynamics of a fluidized bed membrane reactor (FBMR) for catalytic reforming of naphtha in Aspen Plus. The reformer reactor is divided into five sections of plug flow reactors and a continuous stirrer tank reactor with the membrane module to simulate the overall FBMR. Similarly, a fluidized bed reactor (FBR), without membrane permeation phenomenon, is also modelled in the Aspen Plus environment for a comparative study with FBMR. In FBMR, the continuous elimination of permeated hydrogen enhanced the production of aromatics compound in the reformate stream. Moreover, the exergy and economic analyses were carried out for both FBR and FBMR.

## 1. Introduction

Catalytic reforming of naphtha converts low-octane straight-run naphtha, from crude-oil distillation towers, into high-octane reformates. Naphtha reforming has two primary purposes, which are: (1) Production of high-quality octane rating booster to be blended in gasoline streams; and (2) a source of benzene, toluene, xylene isomers (BTX), which are important precursors for further chemical synthesis. As a side effect of the transformations from linear to cycle carbon chain of the hydrocarbon molecules, a considerable amount of hydrogen gas (H_2_) is produced in the process, which is utilized in the refinery (such as in hydrotreating and hydrocracking units) or other applications [1,2].

Although there is a rise in renewable energy resources along with environmental restrictions, the hydrocarbon-based fuel is still widely used, specifically in the transportation sector. The combustion by-products of this fuel have some serious concerns by environmental protection agencies since they are recognized as the leading cause of global warming. To mitigate these effects in the environment, fuel utilization legislations require a high-octane number (ON) for high performance of the combustion process, avoiding delays or mismatches between the optimal combustion process and the movement of the vehicle engines [1]. ON is the quality parameter of gasoline streams that shows how much compression it can withstand without knocking in a gasoline engine. The octane number of gasoline streams is conveniently boosted by naphtha catalytic reforming reformates that occur in three or four radial or axial flow fixed packed bed reactors. It is a fixed bed type of reactor in which the catalyst is placed in a dumped arrangement. Whereas the mode of operation is dependent on the design and is classified as semi-regenerative, cyclic or the newer continuous regenerative type based on the mode of the catalyst regeneration stage. In the naphtha reforming, the catalyst particle size is kept at a value in which there is a compromise between the pressure drop and increased surface area. Larger particles provide less resistance to the gas flow, although they have a low particle effectiveness factor [2].

Considering that the reforming reaction in the equilibrium uses straight-run naphtha molecules to yield the reformate plus H_2_, the desired forward reaction can be boosted by selective removal of the H_2_ from product gases [3,4]. To improve naphtha reforming and pure hydrogen recovery, membrane assisted fluidized bed reactors are recommended [5,6]. In this study, a fluidized catalyst bed reactor is included with Pd membrane-based walls in the naphtha reforming process. This reactor configuration enables the selective in-situ removal of hydrogen from product gases, which increases the production of aromatics. In the membrane reactor, the walls are replaced by a perm-selective membrane material. The reactor design is very important in this regard for maximum yield and simultaneous in-situ hydrogen recovery [7,8].

The developing membrane reactor technology increases hydrogen production and facilitates higher yields of aromatics, as well. In the reported studies, palladium, and its alloys such as palladium-copper [9], palladium-silver [4,10], and only palladium [8] have been used as membrane reactors. For the synthesis of methanol, Rahimpour proposed membrane reactors with the Pd–Ag membrane and pure Pd membrane [11]. Pasha et al. [12] developed FBMR for steam methane reforming in the Aspen Plus environment. Tosti et al. [13] experimented with different configurations of palladium-based membrane reactors for the extraction of ultra-pure hydrogen. Roy et al. [14] worked on the simulation of membrane-based fluidized bed reformers and their economic aspects. Khosravanipour and Rahimpour [15] as well as Rahimpour et al. [11] presented the concept of membrane assisted naphtha reformer and studied the effects of in-situ hydrogen separation in a packed bed reactor and fluidized bed reactor for naphtha reforming. Their results showed an enhancement of aromatics along the reactor and studied the effects of combining the endothermic naphtha reforming reaction and hydrogenation of aniline to nitrobenzene in a thermally coupled fluidized bed reactor. Modelling a membrane reactor is a challenging task due to the simultaneous occurrence of diffusion coupled with the mass transfer and chemical reaction inside the reactor [16].

In naphtha reforming, the studies on FBMR that used mathematical and computing programming languages such as MATLAB or FORTRAN are not readily accessible to the design engineers in the process industry. In this study, a FBMR for naphtha reforming is developed on the Aspen Plus platform. Aspen Plus is a widely employed process simulator for industrial process simulations. In the software, the physical phenomena on the FBMR are implemented utilizing the hydrodynamics theory as an integrated sub-model. Additionally, chemical reactions are conveniently implemented by the built-in power-law input panel of Aspen Plus. Ideal reactor models are available as modules in Aspen Plus and are combined successively to mimic the behaviour inside the fluidized bed membrane reactors [17]. An interface to Excel is used for supplying hydrodynamic parameters to Aspen for calculation of volumes and voidage in CSTR and PFR blocks of Aspen Plus. Naphtha reforming is energy intensive process therefore high energy efficiency is desired to improve its feasibility and sustainability. 

The quest of an energy efficient operation has led to the use of exergy (useable energy) to aid in systems engineering design [18]. The exergy-based analysis provides information on the system that comprises multiple domains and disciplines using energy as a common ground, with irreversibilities taken into account [19]. It incorporates the first and second law of thermodynamics and helps in quantifying and minimizing the effect of irreversibility [20]. The exergy and economic analysis of the FBMR and FBR models are performed separately to have a comparative view of the exergy efficiency and economic viability. The Aspen Plus software has been used intensively for a comparative study of FBMR and FBR models, but the exergy analysis was not included in the comparison [21,22,23]. In this study, the MATLAB based algorithm is used to analyze the exergy efficiency of both FBMR and FBR models.

The paper has been organized as follows. The reforming process is described in Section 2. An industrial setup for a semi-regenerative reformer is taken as an example from the literature, where three packed bed reformers are used. Section 3 details the model building and flow sheeting process in the Aspen Plus environment with Excel interfacing. Results from the simulation are discussed and compared with FBR in Section 4, followed by conclusions in Section 5.

## 2. Process Description

During the naphtha reforming process, the low-octane hydrocarbons are modified to yield a high-value reformate. Typically, a naphtha reformer feed is a mixture composed of the following: with a boiling point from 30 to 90 °C is light naphtha (C5 and C6), 90 to 150 °C is medium–weight naphtha (C7 and C9), and 150 to 200 °C is heavy naphtha (C9 and C12). The straight-run naphtha constitutes 15 to 30 wt% of the crude oil. It is obtained directly from the atmospheric crude oil distillation column. Additionally, it is a mixture of paraffin, naphthenes, and aromatics in the C5– C12 range and the boiling point between 30 and 200 °C.

The reformer operating conditions require that the feed be heated at high temperature (~770 K, 3.7 MPa). Reactions are carried out under high hydrogen partial pressure to reduce catalyst deactivation due to coking. Typically, 3–4 serially connected fixed bed reactors are employed for reforming with inter-stage heating. The feed gas is pre-heated with the heat exchange from the effluent of the last reactor. Heat exchangers are usually of the shell and tube type. As the reforming reactions are endothermic, the effluent from each reactor requires re-heating to compensate for the temperature drop and the related rate of reaction decline.

**Figure 1:**
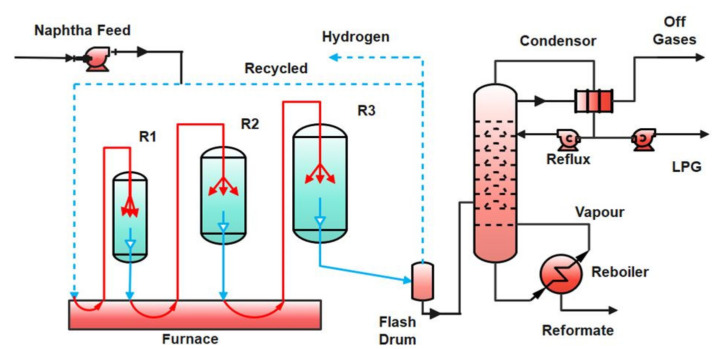
Process flow diagram for the conventional catalytic naphtha reforming process with three reactors (R1, R2, and R3) in series.

As shown in Figure 1, the feed and recycle hydrogen are mixed to attain the desired H_2_/HC ratio and pre-heat the feed stream [11,24]. The pre-heated feed is brought to the reaction temperature of 777 K in the feed heater and is fed to the first reactor. The reactors are loaded with Pt-Re catalysts on an alumina support. The catalyst is bi-functional, where the alumina provides the acid function and Pt-Re provides the metal function for dehydrogenation of naphthenes. The partially reacted effluent from reactor 1 is brought up to the reaction temperature in heater 2 and becomes the feed to reactor 2. With the passage through the reactors, the rates of reaction drop resulting in the increased reactor volume. There is a notable drop in the endothermicity of the reactions and consequently, the heating requirements also decrease. The product stream from the third reactor is first pre-cooled with the incoming feed and then is sent to a flash separator vessel, where the liquid and gaseous components are separated. Cooling of the product stream is required due to its high temperature. A drop in temperature affects the separation of lighter gases from the reformate liquid. The flashed-gas contains hydrogen along with products of cracking, which are mainly a small quantity of light gases such as methane, ethane, propane, and butane. The hydrogen from the flash separator is split into two parts. One part is compressed and added to the naphtha feed to maintain the inlet H_2_/HC ratio. The liquid product from the bottom is sent to the fractionation section (stabilizer).

The dehydrogenation reaction is the main reaction responsible for the rise of the octane number (ON) value [25]. The temperature drops by almost 50 °C in the first reactor, which essentially quenches other reactions and thus requires re-heating of the reactants. To maintain the inlet condition of each reactor, a heat exchanger is used to accommodate the changes in pressure and temperature. A bi-functional catalyst is employed for the reforming process. The two functions are metallic and acidic and are needed for different reactions. Hydrogenation and dehydrogenation reactions are catalyzed by the metal function, while the acid function promotes the isomerization and cyclization reactions [26]. The dehydrogenation reaction, which is the dominant reaction has been studied and reported in the literature. The first reported study is from Smith [27], which included dehydrogenation in his four lumped model. Other variations of Smith’s model have been proposed later. Marin et al. [28], Ramage et al. [29], Jorge and Eduardo [30], Hu et al. [31], Padmavathi and Chaudhuri [32], and Weifeng et al. [33] have performed detailed studies regarding reforming kinetics. The dehydrogenation reaction scheme is presented in Table 1 [6].

## 3. Modelling and Analysis Methods

### 3.1. Membrane Reactor and its Modelling Method

**Figure 2:**
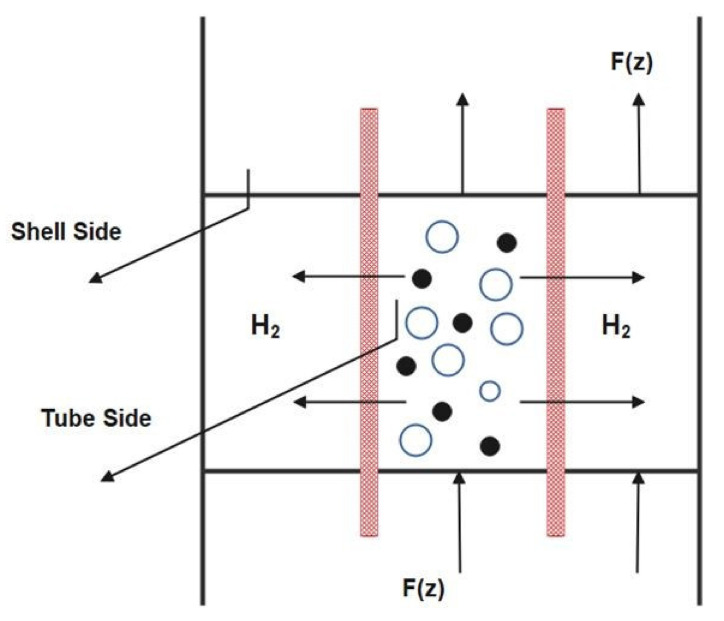
Cross sectional view of Fluidized bed membrane reactor model.

**Figure 3:**
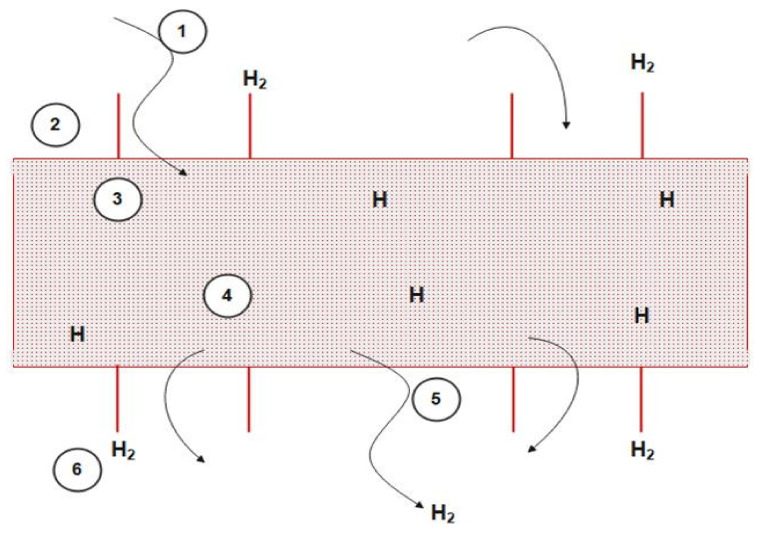
Hydrogen permeation from membrane.

To fluidize a fixed bed, the catalyst particles are crushed to a small size (100 microns). This is found in the FBMR scheme for naphtha reforming, as depicted in Figure 2 [11] “reproduced with permission from M.R. Rahimpour, international journal of hydrogen energy; published by Elsevier, 2009”. During the reforming process, the heat and mass transfer occur within the reactor creating a hydrogen partial pressure gradient that results in a net transfer of hydrogen to the shell side. This transfer of excess hydrogen results in displacing the reaction towards the formation of more product. The fluidization of catalyst particles is carried out by feeding the catalyst filled reactor with gas from the bottom through a porous plate distributor. Hydrogen gas is used as a sweep gas in the shell compartment, where its flow is co-current with the reactant gas. Fluidization results in a very low-pressure drop even using a very small catalyst size, which would not be feasible in a fixed bed. The membrane material of selection is a palladium-silver alloy combining the excellent perm selectivity of palladium with silver providing mechanical stability. Hydrogen gas in the product permeates through the membrane surface. This permeation results in the displacement of equilibrium in the forward direction. In this reactor configuration, reformate and hydrogen production increases as hydrogen is being separated from each reactor. The hydrogen yields consequently decrease owing to the compositional difference with the fixed bed reactor. The hydrogen permeation process is shown in Figure 3 [34] “reproduced with permission from Samhun Yun, journal of membrane sciences; published by Elsevier, 2011”. To control the hydrogen permeation, pressure is used as a driving force in the shell side of each reactor. The thickness of the dense membrane for modelling in Aspen Plus v11.0 is set to 20 µm and is mounted with a stainless-steel support. The membrane’s length is equal to 6.29, 7.13, and 7.89 m and the area is 30.02, 37.39, and 49.05 m^2^ for reactor 1, 2, and 3, respectively [6].

An Excel calculator block integrated into Aspen Plus is developed for the calculation of hydrodynamic parameters, using the two-phase theory of fluidization that calculates the catalyst weight, as well as the distribution and volume of CSTR and PFR combination.

### 3.2. Preliminary Assumptions:

- The dense catalyst bed has two identifiable phases: A bubble phase and an emulsion phase.
- Steady-state and pseudo-steady-state operation is assumed.
- Much of the reactions occur within the emulsion phase.
- Permeation of hydrogen is assumed to occur from the emulsion phase only.
- Hydrogen diffuses through the membrane radially.
- Assumption of spherical bubbles hold.
- The movement of gas in bubbles is assumed to follow the plug flow. Additionally, due to a very low quantity of catalyst, the reaction rates are very low compared to the emulsion gas phase velocity.
- Contents of the bed are well mixed and both emulsion and bubble phases are at a uniform temperature.
- Adiabatic conditions.
- Sieverts’ law is applicable for hydrogen permeation through the membrane [35] (Equation (1)).

(1) $$Q_{H_{2}}=\eta kC_{mp}\left[P_{RH_{2}}^{0.5}-P_{MH_{2}}^{0.5}\right]e^{\left(-\frac{E_{a}}{RT}\right)}\;$$

The Aspen Plus based model simulation of FBMR and FBR is performed and detail results are reported in Section 4. The equations used from the literature are presented in Table 2 [36]. The output from the block is transferred to CSTR and PFR units through an internal Excel interface and transfer modules. A fluidized bed exhibits complex hydrodynamics. To model its behavior, the dense bed is divided into a bubble phase and an emulsion phase. Membrane permeation occurs simultaneously with the reaction. Gas flowing in the form of bubbles is modelled through a plug flow reactor and the emulsion phase is modelled through CSTR. The fluidized bed reactor is represented by PFR and CSTR, which are available standard modules in Aspen Plus. A separate ‘SPLT’ Excel file is used to implement the equations described in Table 2. After estimating the hydrodynamic parameters, the data are transferred to Aspen Plus, which uses its internal database to calculate the thermodynamic properties based on material and energy balance equations. The effluent streams from each section are then transferred to the ‘TRF’ Excel block, where the mass transfer equation (in the case of FBR) and additionally Sievert’s equation (in the case of FBMR) are implemented. Afterwards, the exit streams are transferred to the respective PFR and CSTR for the next section (i+1). Calculations then proceed in this manner until they reach the topmost section of the bed.

The membrane performance is affected by non-uniformity in the membrane fabrication and blockage of the membrane surface by catalyst dust. The membrane permeation effectiveness factor (η) accounts for all the negative influences on the permeation rate and its value is determined experimentally [4]. To simulate the hydrogen permeation process through the membrane tube, a User Model 2-unit operation block with an Excel spreadsheet is used to perform the calculations. Aspen Plus supplies the properties of the feed stream of the user model and some additional parameters ($\eta ,\;k,{\;C}_{\mathrm{mp}},\;E,\;R,\;T,{\;P}_{{\mathrm{RH}}_{2}},{\;\mathrm{and}\;P}_{{\mathrm{MH}}_{2}}$) to the Excel spreadsheet. The additional parameters are shown in Table 2. Excel organizes this information and calculates the product stream properties with the hydrogen production rate ($Q_{H_{2}}$) based on Sieverts’ law. This information is then returned to the Aspen Plus interface and results are displayed. The effect of increasing the number of stages is an increase in the transfer of partially reacted bubble gas to emulsion gas, where higher chances for the reaction exist. The right number of stages to model this system is dependent on its kinetics and hydrodynamics.

### 3.3. Exergy Analysis

Exergy is the maximum amount of work available by bringing the source through the reversible process into its equilibrium with its environment. It is also known as the ability of energy to do valuable work [18]. The maximum amount of output work occurs when the system gains equilibrium reversibly. However, the actual work is quite smaller due to process irreversibility [37]. 

**Figure 4:**
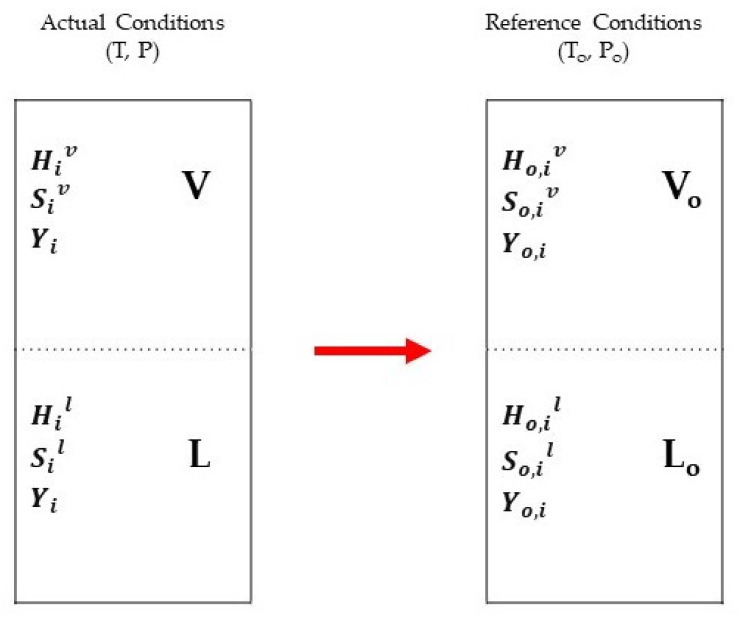
Exergy change from actual to reference conditions.

Physical exergy, considered in this study, of a stream is exergy arising from the difference of the actual temperature and pressure condition *(T, P)* from reference values $\left(T_{o},P_{o}\right)$. As shown in Figure 4, physical exergy represents the thermomechanical part of total exergy [21]. The physical exergy term can be expressed in the equation below.

(2) $$Ex=Ex^{phy}=\Delta_{actual\;\to 0}\left[L\left(\overset{n}{\underset{i=1}{\sum }}\cdot x_{i}H_{i}^{l}-T_{o}\overset{n}{\underset{i=1}{\sum }}\cdot x_{i}S_{i}^{l}\right)+V\left(\overset{n}{\underset{i=1}{\sum }}\cdot x_{i}H_{i}^{v}-T_{o}\overset{n}{\underset{i=1}{\sum }}\cdot x_{i}S_{i}^{v}\right)\right]$$

For the physical exergy calculation, the enthalpy and entropy at a certain reference condition are required. Where $H_{i}^{l}$ and $S_{i}^{l}$ are the enthalpy and entropy in the liquid phase, while $H_{i}^{v}$ and $S_{i}^{v}$ are the enthalpy and entropy in the vapour phase, transition depicted in Figure 4. Additionally, *T_o_* is the temperature at the reference condition.

### 3.4. Economic Analysis

For the economics of a process design, there are three fundamental rules: (a) Estimation of the design options, (b) optimization of the process operations, and (c) overall project profitability. The total cost required for a new project can be divided into five main points.

Battery limit investment: Battery limit denotes the geographic boundary that describes the manufacturing area of a process plant. It encompasses structure, equipment, and buildings. The battery limit investment involves the acquisition of every distinct plant item and its installation that makes up a working process. The cost of certain equipment depends on the capacity, material of construction, design and operating pressure, and temperature of equipment. Cost data can be taken from capacity versus cost charts or power law [38].

(3) $$C_{EQP}=C_{BASE\;}{\left(\frac{Q}{Q_{BASE}}\right)}^{Z}$$

where $C_{EQP}$ is the cost of the equipment with capacity *Q*, $C_{BASE\;}$ is the cost of the equipment with base capacity $Q_{BASE}$, *Z* is constant and depends on the type of equipment, and A is the larger amount of the published data, which is available in the literature. The published data are mostly old, require an update, and can be put on a common basis using indexes [39].

(4) $$\frac{C_{Y1}}{C_{Y2}}=\frac{I_{Y1}}{I_{Y2}}$$

where *C_Y_*_1_ and *C_Y_*_2_ are the costs in the first and second years, respectively. Moreover, *I_Y_*_1_ and *I_Y_*_2_ are the indices in the first and second years, respectively.

Commonly used indices are the Chemical Engineering Indexes and Marshall and Swift, published in the Chemical Engineering magazine, whereas the Nelson–Farrar Cost Indexes for refinery construction are published in the Oil and Gas Journal [38]. The Chemical Engineering Process Cost Index (CEPCI) is widely used in process industries [39]. Finally, the material of construction, the pressure, and the temperature of the design affect the cost of equipment. The capital cost factors for typical material of construction, pressure, and temperature are given in the literature [3,40].

(5) $$C_{EQP}=C_{BASE}{\left(\frac{Q}{Q_{BASE}}\right)}^{Z}\;f_{MAT}\;f_{PRE}\;f_{TFM}$$

where *f_MAT_* is the correction factor for MOC, *f_PRE_* is the correction factor for pressure. Additionally, *f_TEM_* is the correction factor for temperature

Utility investment: Capital cost in utility includes generation and distribution of electricity, process water, refrigeration, and compressed water.

Off-site investment: This includes roads and paths, guardhouses, warehouses, and loading and weighment devices. 

Working capital: The expenses which are invested prior to any production. It includes product inventories, material transportation cost for start-up, and credits extended to customers and suppliers. 

Total capital cost: The total capital cost of the process, services, and working capital can be obtained by applying multiplying factors or installation factors to the purchase cost of individual items of equipment [41]. The capital cost of the Pd-Ag based membrane and stainless-steel frame is taken from the experimental investigations carried out by US-DOE [42].

(6) $$C_{FIX}=\sum f_{I}C_{EQP,\;I}$$

where *C_FIX_* is the fixed capital cost of a complete project.

The installation factor for the new design is broken down in Table 3 into constituent parts, according to the governing phase being processed. Therefore, for the installation factors, the application and estimation of the total capital cost in the following equation is used [42,43].

(7) $$C_{FIX}=\sum {\left[f_{MAT}f_{PRE}f_{TEM}\left(\;1+f_{PIPING}\right)\right]}_{I}\;C_{EQP,I}+\left(\;f_{EQPR}+f_{INSTR}+f_{UTILITY}+f_{OffS}+f_{BUILD}+f_{DECONC}+f_{CONT}+f_{WRKS}\right)\sum C_{EQP,I}$$

## 4. Results and Discussion

### 4.1. FBMR vs. FBR Model Design

**Figure 5:**
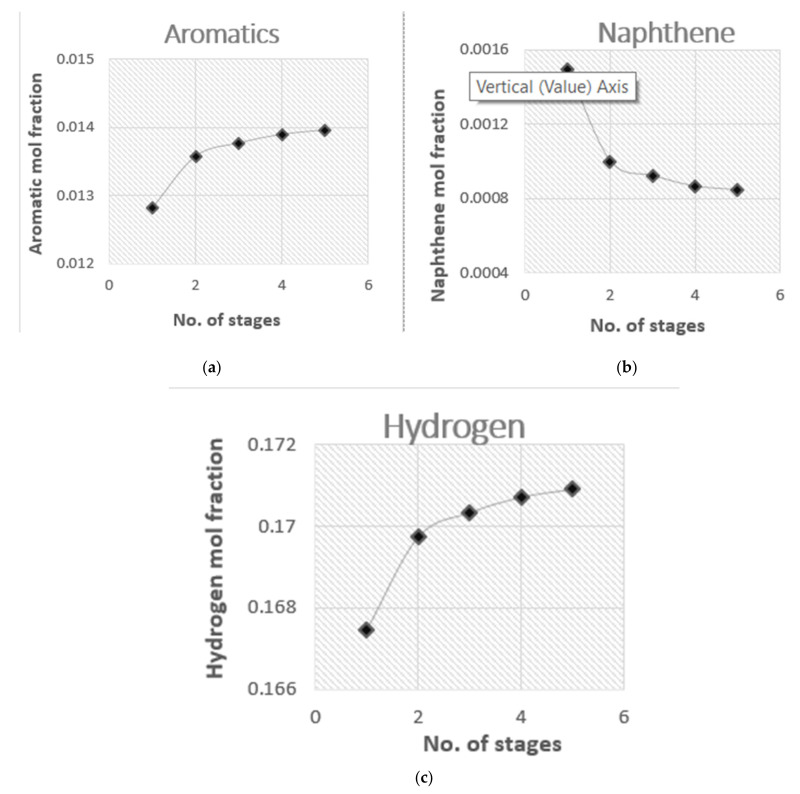
Effect of the number of stages on FBMR for (**a**) aromatics; (**b**) naphthene; (**c**) hydrogen.

**Figure 6:**
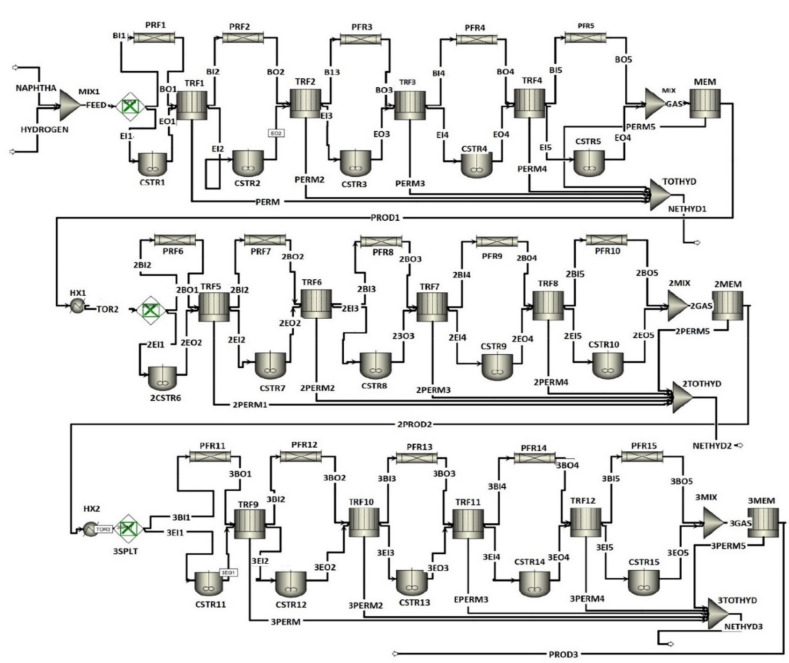
Process flow diagram of FBMR.

**Figure 7:**
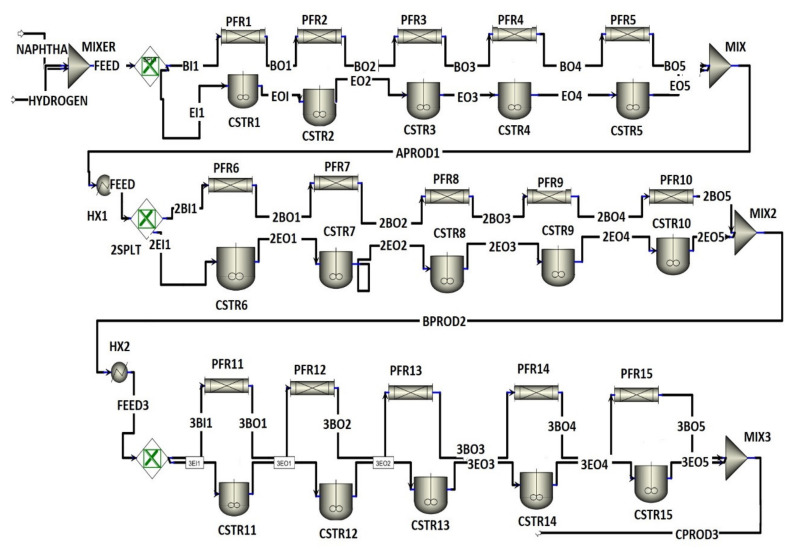
Process flow diagram of FBR.

The reformer is divided into five sections to simulate the conditions inside a real-world operational unit. Figure 5 shows that, as the number of stages is increased, the rate of reaction increases. For the FBMR, the optimum number of stages was determined to be 5. The Aspen Plus based process flow diagram of FBMR and FBR is given in Figure 6 and Figure 7, respectively.

### 4.2. Parametric Analysis

Several variables affect the performance of reactors on FBMR. The more important parameters are the temperature at which the reaction is carried out, the pressure of the shell side, and the hydrogen to hydrocarbon molar ratio.

#### 4.2.1. Influence of Reactor Temperature

**Figure 8:**
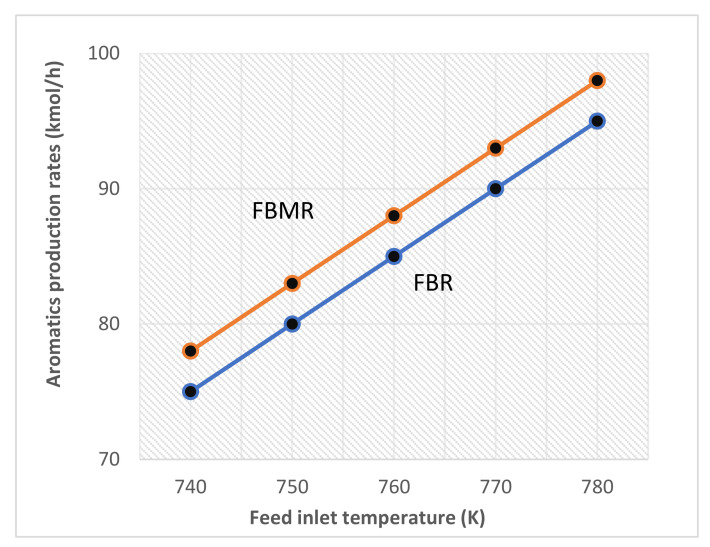
Effect of temperature on aromatic production.

Since the reactions are highly endothermic in nature and thus high temperature favours it. In Figure 8, it is shown that a temperature rise has a favourable impact on the aromatics’ mole fraction. As the reaction proceeds inside the reactor the temperature will drop and the reaction rate will decrease due to its endothermic nature. Therefore, the reaction is carried out in three separate adiabatic reactor vessels with varying catalyst amounts. Additionally, inter-stage heaters are provided to re-heat the product stream to the reaction temperature. A similar trend is mentioned by Rahimpour [6], in which the membrane-based fluidized reactor has increased production than the conventional fluidized reactor.

#### 4.2.2. Influence of Shell-Side Pressure

**Figure 9:**
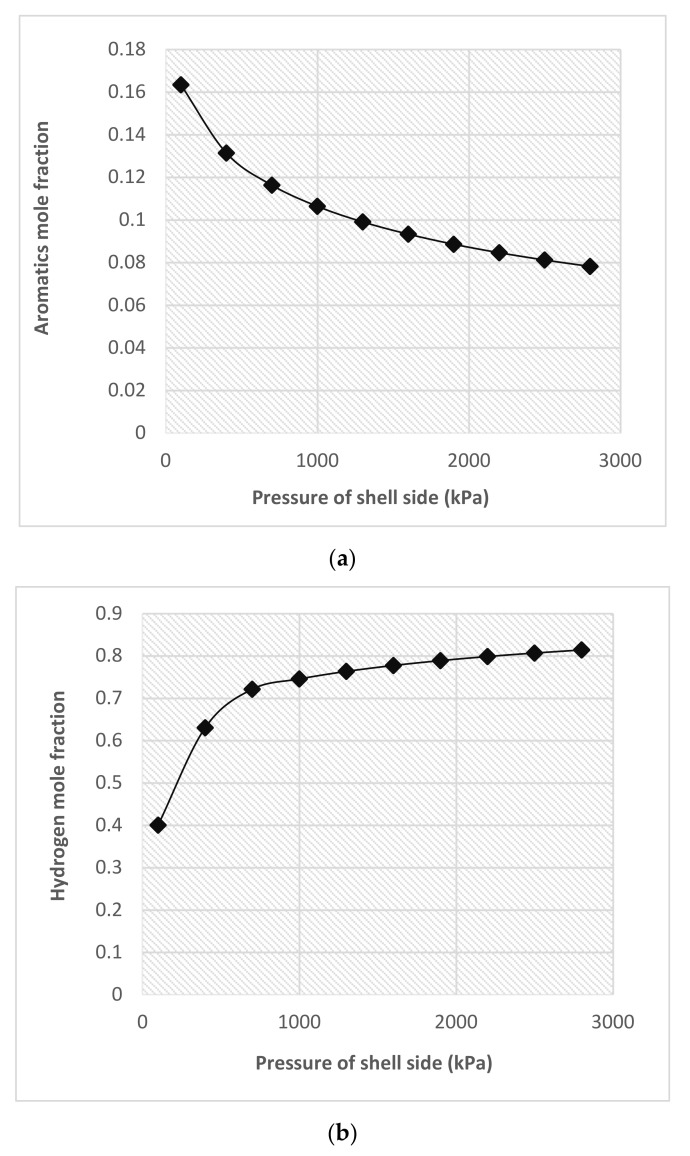
(**a**) Mole fraction of aromatic; and (**b**) mole fraction of outlet hydrogen in the reaction side as a function of shell side pressure.

The difference between the pressure of reaction side and permeate side creates a driving force for hydrogen permeation. As the dehydrogenation reaction is the hydrogen producer, with the reaction proceeding more and more hydrogen will be produced. In the case of FBR, this hydrogen accumulates inside the reactor and increases its partial pressure as well as increases the affinity for products to move towards the left side, i.e., increasing the moles of reactants. Rahimpour [6] showed that in a traditional FBR, the aromatic mole fraction at 2300kPa is equal to 0.043. According to Figure 9a when the pressure of shell side is approximately less than 2300 kPa, the aromatic mole fraction is higher than 0.043. This figure shows that the trend must be followed in shell pressure reduction to have aromatic mole fractions greater than tradiotional FBR. Moreover, in the case of the FBMR, the excess hydrogen is removed alongside the wall, and thus keeps its partial pressure constant or even decreases it if the shell side pressure is further reduced. This is the main reason that the FBMR produces more hydrogen as compared to FBR, due to the increased rate of the forward reaction, as shown in Figure 9b. While the pressure inside the reactor is controlled within narrow limits, the pressure inside the shell is varied. As a result, hydrogen and thus aromatic production are controlled in the FBMR. Wieland et al. [43] showed that the Pd-Ag membrane achieved maximum theoretical recovery of hydrogen on the increased pressure for hydrogen production and recovery through membrane reactors. 

#### 4.2.3. Influence of Membrane Thickness

**Figure 10:**
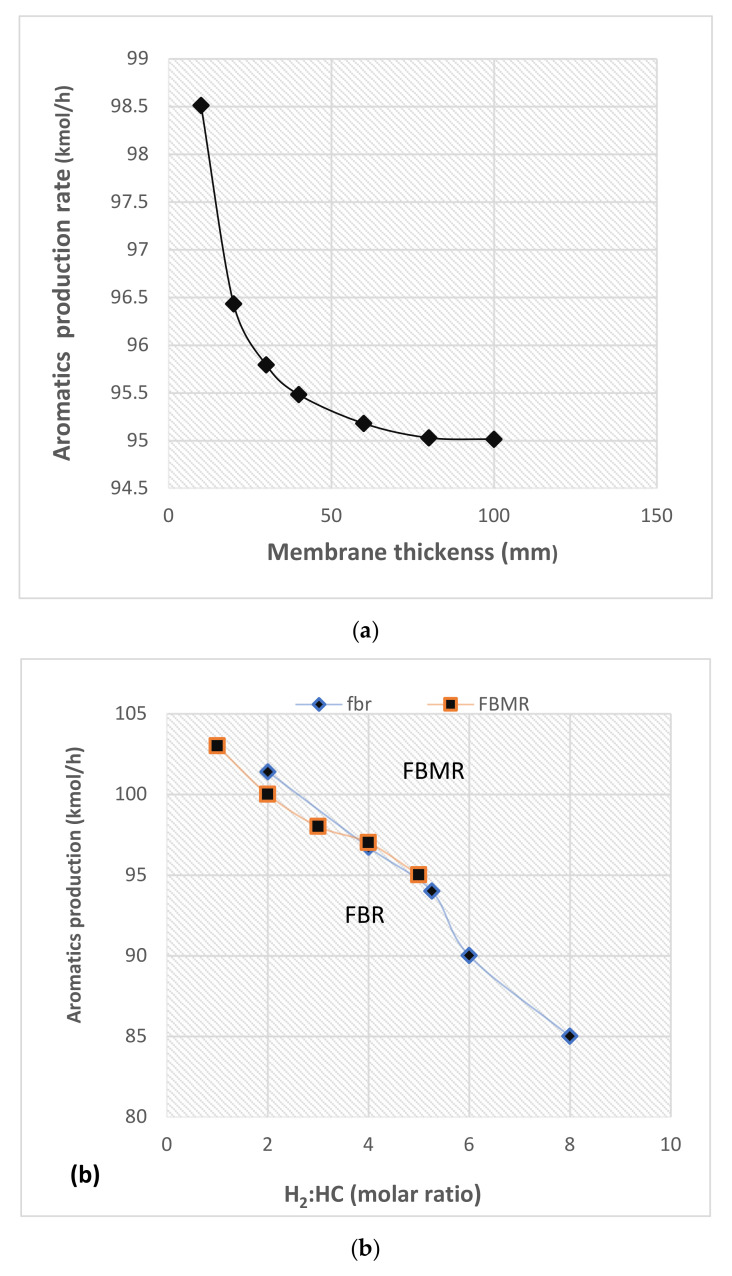
(**a**) Aromatic production as a function of the membrane; (**b**) aromatic production rate vs. H_2_/HC ratio.4.2.4. Influence of H_2_/HC

The effect of membrane thickness on the molar aromatic production is investigated. The result is plotted in Figure 10a. When the membrane is very thin around 10 microns, aromatic production shows a sharp increase with further reduction in thickness. Furthermore, it is observed that when the thickness is about 50 microns, a further increase in thickness does not bring any significant reduction in aromatic molar production. The thin membrane requires a support material. Stainless steel and alumina are the more frequently used materials for providing support. Alloying with silver is also a technique to provide mechanical strength. Tong et al. [44] investigated hydrogen recovery through palladium membranes of different thicknesses and reported that the recovery rate almost gets doubled using the 8-micron membrane comparatively to the 11-micron membrane at the same parametric conditions.

The naphtha reforming reactions proceed under a hydrogen atmosphere to suppress the undesired cracking reactions. The hydrogen to hydrocarbon molar ratio is an important parameter from an industrial standpoint. Therefore, its variation on aromatic production is included in this study. The higher hydrogen to hydrocarbon ratio results in the lower aromatic molar production, which can be seen in Figure 10b. The effect of a high H_2_/HC ratio is more in the case of FBR as compared to FBMR. The higher molar ratio in FBMR is due to the continuous in-situ hydrogen removal, which keeps the aromatic molar flow rate higher than FBR.

### 4.3. Aromatics and Hydrogen Yields

In Table 4, the component-wise yield through the FBR and FBMR is compared. The first column shows the individual hydrocarbon components fed to both reactors. The second column gives the mole fraction of each component, whereas the next column is the product flow rate of FBR (CPROD3) and FBMR (PROD3). In the case of FBMR, part of the hydrogen is continuously removed resulting in a higher production rate of aromatics. Simultaneously, a portion of the pure hydrogen is recycled back to reactors 2 and 3 to maintain the hydrogen to hydrocarbons ratio (H_2_:HC = 5.69:1). The product streams of the FBMR and FBR show a very significant difference of aromatics and reformates production along with off-gases and hydrogen production yields. As per Rahimpour [6], the hydrogen partial pressure is directly related to the hydrogen to hydrocarbon ratio. The low hydrogen to hydrocarbon ratio leads to coke formation. Therefore, the hydrogen to hydrocarbon ratio should be held at a reasonable value, as it is crucial to maintain their molar ratio.

In Table 5, the output from the FBMR and FBR is summarized. The first column shows the quantity of aromatic and hydrogen in the feed. The second and third columns show the production rates of the respective component of the FBR and FBMR system. The calculated daily and yearly increase in aromatic and hydrogen is tabulated and used for the cost estimations of FBR and FBMR configuration.

### 4.4. Thermoeconomic Analysis

The physical and mixing exergy analysis is carried out at a reference temperature and pressure of 25 °C and 101.3 kPa, respectively. The summary of the exergy analysis is provided in Table 6. Mustafa et al. [45] reported a detailed exergy analysis on the fluidized naphtha reforming process. Moreover, the authors mentioned that the mechanical and thermal exergies combine to form the physical exergy and that irreversible losses are mainly due to intertial and viscous resistances. As these resistances are significant in the reactor, a decrease in mechanical exergy is inevitable. In addition, due to the endothermic nature of reforming reactions, the physical exergy of the FBMR reactors decreases. Akram et al. and Mustafa et al. [45,46] reported that most of the reactions in naphtha reforming are endothermic and lead to the decreased temperature, which in turn increases irreversibility. Therefore, due to this fact, irreversible losses result in decreased physical exergy.

The total capital investment and operating cost were calculated utilizing the straight-line depreciation method for a time-horizon of 12 years. The income tax rate and interest rates are 45% and 10%, respectively. The manpower costs are taken at a rate of 0.03%. As per feed and product flow rates by model as well as their market values, annual gross sales are given in Table 7 and Table 8 for FBR and FBMR, respectively.

The detailed work of the Direct Cost (costs for equipment, piping, civil, structural steel, instrumentation and controls, electrical equipment and materials, insulation, and paint), labour cost, chemicals, and catalyst, etc., is performed utilizing factors from Table 3. The CEPCI index for the year 2020 is 589.4. Table 9 shows the detailed equipment cost comparisons of FBR and FBMR. The thermal utility used in FBR and FBMR is the same, which is why both have the same utility cost. Moreover, the working capital is the same for both FBR and FBMR. The Lang factor cost clearly shows the difference between the cost for complete projects. The difference is for FBMR due to the membrane addition, which resulted in the increased cost.

The cost of manufacturing (COM) is calculated by Equation (8), here *C_WT_* is considered as zero.

(8) $$COM=0.18C_{CIL}+2.76C_{OL}+1.23\left(C_{UTILITY}+C_{WT}+C_{RM}\right)$$

*C_OL_* is the cost of operating labour, *C_UTILITY_* is the cost of utility, *C_WT_* is the waste treatment cost, and *C_RM_* is the raw material cost.

**Figure 11:**
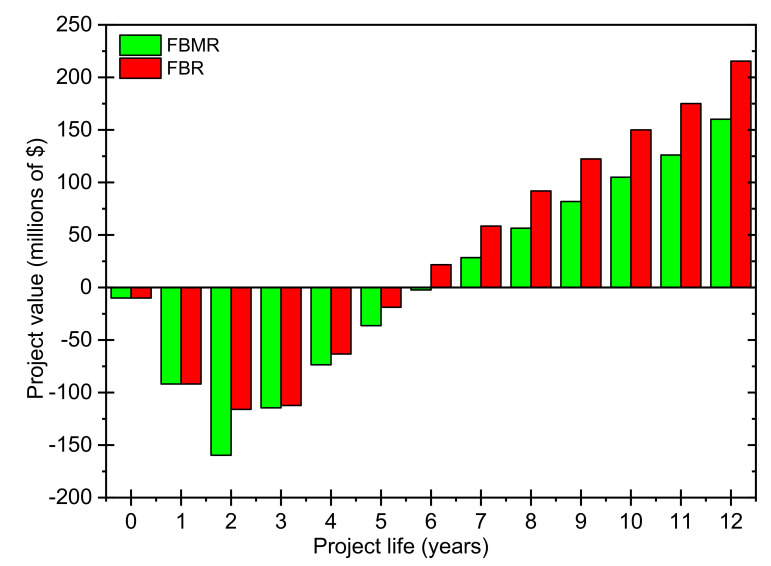
Cumulative cash flow diagram for FBMR and FBR.

For a profitability analysis, discounted cumulative cash flow diagrams for 12 years with an initial construction period of 2 years are constructed, as shown in Figure 11 for FBMR and FBR. The highest discounted cash cumulative cash position, also known as the net present value (NPV), of USD 160.19 million for FBMR and USD 215.48 million for FBR is given in Table 10 and Table 11, respectively. As per the discounted profitability criteria, the payback period of 3.6 and 2.6 years are obtained for both FBMR and FBR, respectively.

## 5. Conclusions

A fluidized bed naphtha reformer with the in-situ membrane separation model is developed in the Aspen Plus environment. The hydrodynamic parameters and membrane permeation phenomena are implemented using Excel interfacing. The results of the fluidized bed membrane reactor (FBMR) are compared with a simple fluidized bed reactor (FBR). It is observed that hydrogen removal from the permeate side drove the reaction forward and resulted in an increase in the aromatic yield. The physical exergy of the outlet stream of FBMR is lesser than that of FBR, due to the higher irreversibility in the reactor. The cost analysis shows that the Lang factor cost is USD 203.36 and 141.74 MM for the FBMR and FBR, respectively. The cost of manufacturing for FBMR and FBR are USD 117.08 and 116.81 MM/year, respectively. Moreover, the net revenue generated by FBMR and FBR is USD 216,754,384 and 234,705,934, respectively. Furthermore, the FBMR and FBR had a payback period of 3.2 and 2.6 years, respectively.

## Figures and Tables

**Table 1:** Dehydrogenation reactions with the rate constant and heat of reaction data.

$ACH↔A_{n}+3H_{2}$	$r_{1n=}k_{1n}\left(P_{ACHn}-\frac{P_{An}P_{H2}^{3}}{K_{1n}}\right)$	$k_{1n}=exp\left(a-\frac{E}{RT}\right)$ $\left(kmol.kg_{cat}^{-1}.h^{-1}.kPa^{-1}\right)$		$K_{1n}=exp\left(A-\frac{B}{T}\right)$ ${\left(kPa\right)}^{3}$	
	$\Delta H\left(\frac{kJ}{molH_{2}}\right)$	**a**	$\frac{E}{R}\times 10^{-3}$	**A**	**B**
C_6_	68.73	18.75	19.50	59.90	24,800
C_7_	208.47	20.70	19.50	60.23	25,080
C_8_					
*for* A_n_ = MX *	64.50	17.89	19.50	60.37	23,270
*for* A_n_ = OX *	65.10	19.15	19.50	60.32	23,490
*for* A_n_ = PX *	64.74	18.66	19.50	60.13	23,360
*for* A_n_ = EB *	68.70	18.71	19.50	60.40	24,780
C9+	66.05	20.38	19.50	61.05	21,330

* Improvements made to the Padmavathi et al. model.

**Table 2:** Parameters for hydrodynamic calculation>.

Studied Parameter	Model Equation
Superficial velocity at minimum fluidization	$\frac{1.75}{\epsilon_{mf}^{3}\varphi_{s}}{\left[\frac{d_{p}\rho_{g}u_{mf}}{\mu }\right]}^{2}+\frac{150\left(1-\epsilon_{mf}\right)}{\epsilon_{mf}^{3}\varphi_{s}}\left[\frac{d_{p}\rho_{g}u_{mf}}{\mu }\right]=Ar$
Archimedes’ number	$Ar=\frac{d_{p}^{3}\rho_{g}\left(\rho_{p}-\rho_{g}\right)g}{\mu^{2}}$
Bubble diameter	$d_{b}=d_{bm}\left(d_{bm}-d_{b0}\right)exp\left(-0.3z/D\right)$ $d_{b0}=0.376{\left(u_{0}-u_{mf}\right)}^{2}$ $d_{bm}=0.65{\left[\frac{\pi }{4}D^{2}\left(u_{0}-u_{mf}\right)\right]}^{0.4}$
Coefficient of mass transfer (Bubble to emulsion phase)	$K_{be}=\frac{u_{mf}}{3}{\left[\left(\frac{4D_{jm}\epsilon_{mf}u_{b}}{\left(\pi d_{b}\right)}\right)\right]}^{1/2}$
The velocity of bubble rise	$u_{b}=u-u_{mf}+0.711\sqrt{gd_{b}}$
The volume fraction of the bubble phase to the overall bed	$\delta =\frac{\left(u-u_{mf}\right)}{u_{b}}$
Specific surface area for bubble	$a_{b}=\frac{6\delta }{d_{b}}$
Density for emulsion phase	$\rho_{e}=\rho_{p}\left(1-\epsilon_{mf}\right)$

**Table 3:** Installation factors for the capital cost of equipment.

Capital Cost for Fluid Processing	
Item	Factor
Direct costs	
Equipment delivered cost	1
Equipment erection, fEQPR	0.4
Piping (installed), fPIPING	0.7
Instrumentation and controls (installed), fINSTR	0.2
Electrical (installed), fELEC	0.1
Utilities, fUTILITY	0.5
Off-sites, fOffS	0.2
Buildings (including services), fBUILD	0.2
Site preparation, fSiteP	0.1
The total capital cost of installed equipment	3.4
**Indirect costs**	
Design, engineering, and construction, fDECONC	1
Contingency (about 10% of fixed capital costs), fCONT	0.4
Total fixed capital cost	4.8
**Working capital**	
Working capital (15% of the total capital cost), fWRKC	0.7
Total capital cost, f I	5.8

*f_I_*: Installation factor. *C_EQP,I_*: Cost of *i*^th^ equipment.

**Table 4:** Aromatics production in fluidized bed reactor and fluidized bed membrane reactor.

Components	FEED	FBR (CPROD3)	FBMR (PROD3)
	Mole Fractions	kmol/h	kmol/h
METHA-01	0.0089	17.14	202.537
ETHAN-01	0.0098	18.76	66.443
PROPA-01	0.0085	16.41	16.405
N-BUT-01	0.0045	8.60	8.590
ISOBU-01	0.0031	5.94	5.947
N-PEN-01	0.0015	2.84	2.836
2-MET-01	0.0032	6.18	6.178
N-HEX-01	0.0097	15.04	0.026
2-MET-02	0.0098	15.34	0.028
N-HEP-01	0.0124	19.11	0.032
2-MET-03	0.0133	20.65	0.036
N-OCT-01	0.0101	9.21	0.001
2:2:4-01	0.0143	13.53	0.002
N-NON-01	0.0066	10.01	0.014
2:2:5-01	0.0103	15.90	0.026
CYCLO-01	0.0033	0.91	0.072
METHY-01	0.0036	0.88	0.077
ETHYL-01	0.0049	0.31	0.012
N-PRO-01	0.0008	0.19	0.017
CYCLO-02	0.0000	0.08	0.081
METHY-02	0.0013	2.43	2.383
ETHYL-02	0.0028	5.25	5.091
N-PRO-02	0.0036	6.79	6.569
N-BUT-02	0.0005	0.97	0.938
BENZE-01	0.0036	19.40	205.080
TOLUE-01	0.0046	24.28	0.773
M-XYL-01	0.0006	9.01	5.561
O-XYL-01	0.0007	8.80	2.448
P-XYL-01	0.0015	12.45	0.679
ETHYL-03	0.0009	10.08	0.553
N-PRO-03	0.0011	9.97	0.065
HYDRO-01	0.8403	1866.82	595.781

**Table 5:** The net increment in aromatics and hydrogen.

Components	Feed (kg/h)	FBR	FBMR	Increase Using the Membrane	
		Out (kg/h)	Out (kg/h)	Daily Increase (kg/day)	Yearly Increase (kg/Y)
**Hydrogen**	3250	14,913.65	17,080.07	2166.42	790,743
**Aromatics**	2374	4182.849	4267.602	84.753	30,934.84

**Table 6:** Exergy analysis for FBR and FBMR.

FBR							
Stream Name	T (K)	P (KPa)	HR	SR	HS	SS	Physical Exergy
FEED	780	3702.991	−26.7816	−0.08856	5.069967	−0.05384	11,468.25
PROD1	770.025	3702.991	−7.69063	−0.03791	15.70902	−0.02079	12,726.43
PROD2	775.9731	3604.925	−4.9959	−0.02995	17.30305	−0.01549	13,092.23
PROD3	777.4178	3506.858	−4.3791	−0.02766	17.4909	−0.01418	13,129.03
FBMR							
**Stream Name**	**T** **(K)**	**P** **(KPa)**	**HR**	**SR**	**HS**	**SS**	**Physical Exergy**
FEED	780	3702.991	−26.7816	−0.08856	5.069967	−0.05384	11,468.25
NETHYD1	776.9028	2800	0.000873	−1.46 × 10^−06^	14.05493	0.000402	30.9085
NETHYD2	776.9891	900	0.000873	−1.46 × 10^−06^	14.0306	0.009842	2540.903
NETHYD3	777.0001	900	0.000873	−1.46 × 10^−06^	14.03092	0.009842	2122.787
PROD1	776.4373	3702.991	−5.83885	−0.03361	17.30526	−0.01722	13,053.95
PROD2	776.9417	3604.925	−7.23389	−0.04482	19.21282	−0.02147	9810.164
PROD3	777.0003	3506.858	−12.8969	−0.07652	21.80172	−0.03476	7016.567

HR= Enthalpy at reference state, SR= Entropy at reference state, HS= Enthalpy in stream condition, SS= Entropy in stream condition

**Table 7:** Gross annual sales of FBR.

Gross Annual Sales of FBR			
Material Name	Price ($/kg)	Flow Rate (kg/h)	Annual Cost
Naphtha	0.30	28,178.00	70,349,195
Natural gas	0.00254	4790.505	79,733
Gasoline	0.90	6523.001	48,855,965
Aromatics	0.99	14,913.65	123,125,549
Hydrogen	1.80	4182.849	62,644,687

**Table 8:** Gross annual sales of FBMR.

Gross Annual Sales of FBMR			
Material Name	Price ($/kg)	Flow Rate (kg/h)	Annual Cost
Naphtha	0.30	28,178.00	70,349,195
Natural Gas	0.00254	7,466.072	155,330
Gasoline	0.90	1,596.609	11,953,721
Aromatics	0.99	17,080.07	140,718,362
Hydrogen	1.80	4,267.602	62,926,971

**Table 9:** Cost comparison of FBMR and FBR.

Cost	FBMR	FBR
Equipment cost	$42,904,400	$29,904,400
Bare module cost	$65,575,400	$45,575,400
Total module	$77,378,972	$53,778,972
Total grass root cost	$77,550,922	$53,950,922
Lang factor	4.74	4.74
Lang factor cost	$203,366,856	$141,746,856
Raw materials costs	$70,349,195	$70,349,195
Working capital	$22,100,000	$22,100,000
Cost of utilities	$866,000	$866,000
Cost of operating labor	$802,920	$802,890
Cost of manufacturing	$117,079,279	$116,810,749
Revenue from Sales	$216,754,384	$234,705,934

**Table 10:** Profitability criteria for FBMR.

Discounted Profitability Criteria	
Net Present Value (millions)	160.19
Discounted Cash Flow Rate of Return	26.17%
Discounted Payback Period (years)	3.2
**Non-Discounted Profitability Criteria**	
Cumulative Cash Position (million)	465.31
Rate of Return on Investment	31.02%
Payback Period (years)	2.5

**Table 11:** Profitability criteria for FBR.

Discounted Profitability Criteria	
Net Present Value (millions)	215.48
Discounted Cash Flow Rate of Return	30.24%
Discounted Payback Period (years)	2.6
**Non-Discounted Profitability Criteria**	
Cumulative Cash Position (million)	582.09
Rate of Return on Investment	38.81%
Payback Period (years)	2.1

## Data Availability

Not applicable

## Nomenclature

Ac	cross section of reactor, m^2^
CSTR	continuous stirred tank reactor
db	bubble diameter, m
Ei	activation energy for the i^th^ reaction, kJ/kmol
Ep	activation energy of permeability, kJ/mol
FBP	final boiling pint (°C)
FSH_2_	flow rate of H_2_ in shell side gas, kmol/h
Ft	total molar flow rate, kmol/h
IBP	initial boiling point, °C
RON	research octane number
kci	coefficient for mass transfer of specie i, m/h
Kei	equilibrium coefficient
kfi	forward rate constant
L	length of reactor, m
MR	membrane reactor
pi	partial pressure of specie i, kPa
Pt	total pressure, kPa
pRH_2_	reaction side hydrogen partial pressure, Pa
pS H_2_	shell side hydrogen partial pressure, Pa
P	permeability of hydrogen through Pd–Ag layer, mol/m^2^ s Pa^0^.^5^
P_0_	pre-exponential factor of hydrogen permeability, mol/m^2^ s Pa^0^.^5^
PBR	packed bed reactor
PFR	plug flow reactor
R	gas constant, kJ/kmol K
ri	rate of reaction for the i reaction, kmol/kg cat h
T	temperature of gas phase, K
TBP	true boiling point, °C
t	time, h
ub	velocity of rise of bubbles, m s^−1^
Cmp	membrane permeation capacity (membrane surface area/thickness)
Ep	activation energy for permeation, J mol^−1^
k	pre-exponential factor, mol km^−1^ h^−1^ Pa^−0^.^5^
Greek letters	
αH	hydrogen permeation rate constant, mol/m s Pa^0^.^5^
d	thickness of palladium layer, mm
ρb	catalyst bed density, kg/m^3^
ρg	density of gas phase, kg/m^3^
vij	stoichiometric coefficient of specie i in reaction j
∆H	heat of reaction, kJ/kmol
ɛb	void fraction of catalyst bed
ɛmf	void fraction of catalytic bed at minimum fluidization
φ	catalyst particle shape factor
δ	fraction of gas in bubble phase
ƞ	permeation effectiveness factor
